# Genomic epidemiology of age-associated meningococcal lineages in national surveillance: an observational cohort study

**DOI:** 10.1016/S1473-3099(15)00267-4

**Published:** 2015-12

**Authors:** Dorothea M C Hill, Jay Lucidarme, Stephen J Gray, Lynne S Newbold, Roisin Ure, Carina Brehony, Odile B Harrison, James E Bray, Keith A Jolley, Holly B Bratcher, Julian Parkhill, Christoph M Tang, Ray Borrow, Martin C J Maiden

**Affiliations:** a Department of Zoology, University of Oxford, Oxford, UK; b Sir William Dunn School of Pathology, University of Oxford, Oxford, UK; c Meningococcal Reference Unit, Public Health England, Manchester Royal Infirmary, Manchester, UK; d Scottish Haemophilus, Legionella, Meningococcus and Pneumococcus Reference Laboratory, Glasgow Royal Infirmary, Glasgow, UK; e Wellcome Trust Sanger Institute, Cambridge, UK

## Abstract

**Background:**

Invasive meningococcal disease (IMD) is a worldwide health issue that is potentially preventable with vaccination. In view of its sporadic nature and the high diversity of *Neisseria meningitidis*, epidemiological surveillance incorporating detailed isolate characterisation is crucial for effective control and understanding the evolving epidemiology of IMD. The Meningitis Research Foundation Meningococcus Genome Library (MRF-MGL) exploits whole-genome sequencing (WGS) for this purpose and presents data on a comprehensive and coherent IMD isolate collection from England and Wales via the internet. We assessed the contribution of these data to investigating IMD epidemiology.

**Methods:**

WGS data were obtained for all 899 IMD isolates available for England and Wales in epidemiological years 2010–11 and 2011–12. The data had been annotated at 1720 loci, analysed, and disseminated online. Information was also available on meningococcal population structure and vaccine (Bexsero, GlaxoSmithKline, Brentford, Middlesex, UK) antigen variants, which enabled the investigation of IMD-associated genotypes over time and by patients' age groups. Population genomic analyses were done with a hierarchical gene-by-gene approach.

**Findings:**

The methods used by MRF-MGL efficiently characterised IMD isolates and information was provided in plain language. At least 20 meningococcal lineages were identified, three of which (hyperinvasive clonal complexes 41/44 [lineage 3], 269 [lineage 2], and 23 [lineage 23]) were responsible for 528 (59%) of IMD isolates. Lineages were highly diverse and showed evidence of extensive recombination. Specific lineages were associated with IMD in particular age groups, with notable diversity in the youngest and oldest individuals. The increased incidence of IMD from 1984 to 2010 in England and Wales was due to successive and concurrent epidemics of different lineages. Genetically, 74% of isolates were characterised as encoding group B capsules: 16% group Y, 6% group W, and 3% group C. Exact peptide matches for individual Bexsero vaccine antigens were present in up to 26% of isolates.

**Interpretation:**

The MRF-MGL represents an effective, broadly applicable model for the storage, analysis, and dissemination of WGS data that can facilitate real-time genomic pathogen surveillance. The data revealed information crucial to effective deployment and assessment of vaccines against *N meningitidis*.

**Funding:**

Meningitis Research Foundation, Wellcome Trust, Public Health England, European Union.

## Introduction

Invasive meningococcal disease (IMD) caused by *Neisseria meningitidis* and encompassing meningitis and severe sepsis has been reported at varying rates worldwide for more than 200 years. *N meningitidis* is ordinarily a harmless commensal of the oropharynx. Disease incidence and asymptomatic carriage rates are age specific. IMD is generally sporadic, with endemic rates being fewer than three cases per 100 000 of the general population, but hyperendemic outbreaks, intense localised outbreaks, and epidemics do occur. These fluctuations are thought to be due to interactions between *N meningitidis* biology, human susceptibility, and, possibly, evolution and spread of particularly invasive strains.^1^ Almost all IMD is caused by *N meningitidis* expressing the polysaccharide capsules that define serogroups A, B, C, W, X, and Y. Multilocus sequence typing (MLST) has shown that most disease is caused by temporally stable hyperinvasive lineages that correspond to specific clonal complexes defined by MLST.^2^

**Figure 1:**
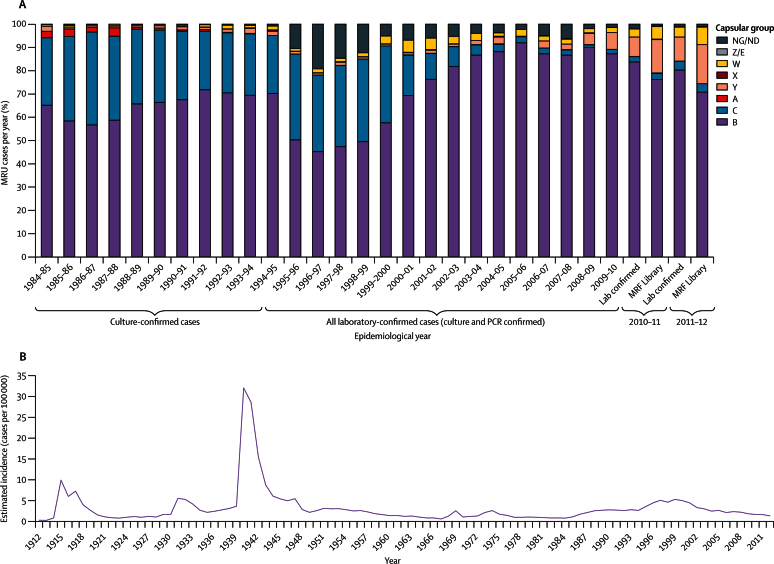
Epidemiology of meningococcal disease in England and Wales, 1912–2012 (A) Proportion of meningococcal serogroups identified from culture and non-culture specimens for epidemiological years 1984–85 to 2011–12. Serogroups were ascertained by serology (for isolates), PCR-based genotyping, or genome sequencing (for the Meningitis Research Foundation Meningococcus Genome Library). (B) Incidence of meningococcal disease. Incidence is calculated on the basis of notification data from Mary Ramsay Public Health England for 1912–97 and laboratory reports of Public Health England for 1998–2012. MRU=Meningococcal Reference Unit. Lab=laboratory. MRF=Meningitis Research Foundation. NG/ND=non-groupable or not determined.

Control of IMD caused by several serogroups has been achieved in the UK by vaccination, such as meningococcal serogroup C conjugate vaccines, which led to herd immunity and a striking reduction in serogroup C disease (figure 1).^3^ Because of poor immunogenicity and safety concerns, however, there are no equivalent vaccines against serogroup B polysaccharides. Serogroup-B-substitute vaccines are based on proteins and often include outer-membrane vesicles.^4^ Vaccine coverage, therefore, depends on serological cross-reactivity. As such vaccines are introduced, comprehensive IMD surveillance is necessary to monitor antigenic variants, their expression, and their association with hyperinvasive lineages.

IMD has been reportable in England and Wales since 1912. Even when alterations in reporting definitions are taken into account, wide fluctuations in incidence are evident (figure 1). IMD reporting encompasses meningitis and septicaemia (the latter diagnosis referring to severe sepsis). Due to antibiotic therapy before microbiological culture from clinical specimens, around half of isolates are culture-confirmed cases (CCC). Linkage with epidemiological data shows that for clinically diagnosed cases in England and Wales the Public Health England (PHE) Meningococcal Reference Unit (MRU) receives 98% of all samples, including isolates for CCC and specimens for non-culture-confirmed cases (NCCC).^5^, ^6^ Isolates are routinely characterised by serological and single-gene-sequencing technologies.^5^

**Research in context**

**Evidence before this study**

We searched PubMed with the terms “*Neisseria meningitidis*”, “genome sequence”, and “epidemiology” to identify reports published in any language before the start of the April, 2014. We identified four studies that have reported the use of whole-genome-sequencing (WGS) data for retrospective resolution of single lineage *N meningitidis* outbreak or hyperendemic scenarios and various case studies in which clinical isolates were used retrospectively for strain characterisation. In three studies, WGS was done for 16–108 meningococcal isolates to investigate meningococcal lineage structure with universally present genes. In two of these studies, this approach revealed robust phylogenetic relationships between isolates of different clonal complexes, which contrasted with poor within-clonal complex resolution. In addition to PubMLST.org, which we used in this study, two publically available *N meningitidis* comparative genomic databases existed, both of which included only previously published genomes and neither of which provided integrated clinical characterisation of strains or strain relationships.

**Added value of this study**

The Meningitis Research Foundation Meningococcus Genome Library (MRF-MGL) adds to the application of WGS in public health microbiology by providing an infrastructure to process, analyse, and interpret bacterial data in clinical settings. It provides a framework capable of real-time analyses of such data for diagnosis or for investigation of outbreaks of meningococcal disease in particular and other bacterial diseases in general. Combining the data in the MRF-MGL with previously published data by use of the in-built data analysis tools of PubMLST.org, we showed the importance of meningococcal lineages to changes in meningococcal epidemiology over time.

**Implications of all the available evidence**

Several reports have made use of the MRF-MGL data in taxonomic, comparative genomic (eg, core-genome delineation), and vaccine antigen characterisation. Data in the MRF-MGL form an invaluable evidence base for vaccination policy. For example, in combination with existing epidemiological evidence and WGS sequences, the data informed the introduction of meningococcal serogroup A, C, W, and Y conjugate vaccines into the UK immunisation schedule.

The potential of whole-genome sequencing (WGS) for bacterial characterisation in clinical microbiology, transmission analyses, and studies of vaccine intervention has been shown in several studies and case studies.^7^, ^8^, ^9^, ^10^, ^11^, ^12^, ^13^, ^14^ For this potential to be realised in routine surveillance, however, sustainable, transparent, and compatible infrastructures are needed for data storage, analysis, and reporting. The Meningitis Research Foundation Meningococcus Genome Library (MRF-MGL) presents such a framework. We used WGS data from all English and Welsh IMD isolates submitted to the PHE-MRU in 2 epidemiological years to investigate IMD epidemiology.

## Methods

### Specimen collection

We used WGS data for all 899 IMD isolates submitted to PHE-MRU over the epidemiological years (July 1 to June 30) 2010–11 (501 isolates, 48% of laboratory-confirmed cases) and 2011–12 (398 isolates, 52% of laboratory-confirmed cases; table 1). In agreement with the PHE Vaccine Preventable Invasive Bacterial Infections Forum, data on serogroup, year, and UK region of isolation were made publicly available for each isolate and other demographic data were available from the PHE-MRU on request.

### Procedures

Isolate culture, DNA extraction, whole-genome sequencing, and de-novo draft-genome assembly were done with validated procedures described previously (appendix).^7^, ^8^, ^15^ Short-read sequences were deposited at the European Nucleotide Archive and assemblies were deposited in the MRF-MGL *Neisseria* Sequence Typing database,^16^, ^17^ both of which have open access. Assemblies were annotated for 1720 locus entries defined and indexed with MRF-MGL *Neisseria* Sequence Typing database numbers (NEIS),^8^, ^15^ including those defining genogroup, typing antigens, and vaccine antigens, and the 1605 full-length loci belonging to the *N meningitidis* core-genome MLST scheme (version 1.0).^15^ Features automatically reported by the database included antigen variants, MLST sequence types and clonal complexes, and ribosomal MLST sequence types based on 49 *N meningitidis* loci.^18^ Single MLST locus sequences were interrupted by the ends of contiguous sequences in eight genomes: these were individually sequenced at the PHE-MRU to complete MLST profiles.

### Genomic analysis

Population genomic analyses were done with a hierarchical gene-by-gene approach^15^, ^19^ that enabled rapid, reproducible visualisation of genome similarity across the entire dataset.^7^, ^19^ The Bacterial Isolate Genome Sequence Database^16^ Genome Comparator tool was used to generate allele-based distance matrices that were visualised as Neighbor-Net diagrams. This approach accounted for the high rates of recombination in meningococcal populations, without discarding variant loci deemed to be generated by recombination.^19^ Loci missing in isolates due to absence in the genome or incomplete assembly were ignored in pairwise comparisons. Maximum-likelihood trees were reconstructed from nucleotide sequence data extracted from the MRF-MGL (appendix). For comparison of antigenic variation, we calculated the proportions of aminoacids at which two compared sequences differed (*p* distances) in antigen peptide sequences fHbp, NHBA, PorA, and NadA^4^ of meningococcal group B vaccine (Bexsero, GlaxoSmithKline, Brentford, Middlesex, UK), with complete deletion of alignment gaps.

### Statistical analysis

Between-year relative risk ratios (RRRs) with 95% CIs (Wald estimates)^20^ were calculated with the epitools package, with the function epitab. Standard R functions were used to do χ^2^ and Fisher's exact tests. Human population data were obtained in October, 2013, from the UK Office for National Statistics for 1996–2012 and from The National Archives for 1912–95. For the age-structured analysis, patients' ages were provided by the PHE-MRU. All statistical analyses were done in R (version 2.1.51) with α set at 0·05 (appendix).

### Role of the funding source

The funders of the study had no role in study design, data collection, data analysis, data interpretation, or writing of the report. The corresponding author had full access to all data in the study and had final responsibility for the decision to submit for publication.

## Results

**Figure 2:**
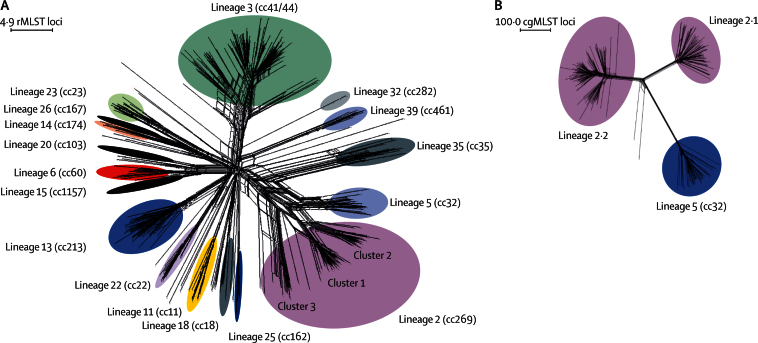
Genetic diversity of disease-associated meningococcal isolates in the Meningitis Research Foundation Meningococcus Genome Library for England and Wales (A) Neighbor-Net graph showing the relationships of all 498 rMLST profiles (ribosomal sequence types) within the 899 isolates available for epidemiological years 2010–11 and 2011–2012. (B) Relationships among isolates belonging to lineage 2 (cc269; n=171) and lineage 5 (cc32) isolates (n=42) assessed with 1605 cgMLST loci. This analysis illustrates the improved resolution achieved with cgMLST for the substructures within and between lineages, compared with rMLST. rMLST=ribosomal multilocus sequence typing. cc=clonal complex. cgMLST=core-genome multilocus sequence typing.

The 899 genome assemblies each contained an average of 209 contiguous sequences and 1571 (98%) completely sequenced core-genome loci, which corresponded to around 79% of the 2000 coding sequences anticipated for each isolate (appendix).^15^ The number of alleles at each locus ranged from two (NEIS2089 and NEIS0627, hypothetical proteins) to 313 (NEIS0829, putative membrane protein). The genomes comprised 272 sequence types, of which 219 were assigned to 20 MLST-defined clonal complexes, and 498 ribosomal sequence types, with 56 from isolates not designated to a known clonal complex (appendix). *N meningitidis* belonging to the hyperinvasive clonal complexes 41/44 (lineage 3), 269 (lineage 2), and 23 (lineage 23) were responsible for 528 CCC (59%), and nine clonal complexes were rare (fewer than ten members). Clonal complexes varied in diversity (figure 2, appendix).

Capsule groups were deduced from WGS-derived sequence data to provide a genogroup for each isolate. These were concordant with PHE-MRU serogroup designations and PCR-derived genogroups.^5^ WGS data provided additional discrimination of the following: two non-groupable isolates, one possessing a capsule null locus and the other capsule E loci; six isolates that were non-groupable because loci were in phase-variable off status; four isolates that were assigned to serogroup and genogroup W/Y^21^ that possessed serine residues at codon 310 of the capsule polymerase, which would result in mixed galactose-containing or glucose-containing sialic acid capsules; and two serogroup B isolates that contained both genogroup B and E loci, although neither showed evidence of serogroup E expression.

668 isolates (74%) were genogroup B, 141 (16%) were genogroup Y, 56 (6%) genogroup W, and 27 (3%) genogroup C, with an additional three genogroup E isolates, one serogroup A isolate, one serogroup X isolate, and one capsule null isolate. Serogroup characteristics of isolates in CCC were similar to those in NCCC, with a slight over-representation of group Y (6%, p<0·0001) and group W (2%, p<0·0001) in the CCC (figure 1). The proportion of CCC in older patients was slightly higher than that for NCCC. These differences were consistent with known differences in clinical presentation and diagnosis in different demographic groups.^22^, ^23^ The differences in serogroups and the proportion laboratory confirmed were similar in the two epidemiological years (table 1).

High-resolution comparisons of isolates were achieved by hierarchical analysis with ribosomal MLST^18^ and core-genome MLST,^15^ as has been described previously,^19^ which enabled visualisation of lineages and their structures (figure 2, appendix). To take advantage of this high resolution, we used a proposed lineage nomenclature to achieve rapid identification of lineage membership by manually linking ribosomal sequence types to lineage and sublineage designations. Sublineages have so far been defined by visual inspection and comparison of phenotypes, such as the ET-15 and non-ET-15 subclusters of clonal complex 11 (lineage 11), which, respectively, are named lineage 11.2 and lineage 11.1.^7^, ^24^ Maximum-likelihood trees generated from variable sites in ribosomal and core-genome MLST loci identified the same lineages and sublineages as allele-based analyses (appendix). Congruence was high for ribosomal sequence types and core-genome MLST relationships, with one exception: lineage 2 sublineages were probably affected by recombination of some ribosomal MLST loci (figure 2, appendix).

In the two epidemiological years, nearly a quarter (average 102 isolates [23%]) of CCC were from individuals younger than 1 year, with the highest incidence seen in patients aged 4–6 months (average 35 isolates [8%]). The age characteristics for MRF-MGL isolates reflected those for NCCC, except for isolates from individuals older than 30 years, in whom the proportion of CCC was slightly greater (an additional 0·01–1·07% for most of the 2 year age groups). The age distribution changed from 2010–11 to 2011–12, with a substantial reduction being seen in cases from individuals younger than 5 years but a significant increase in cases from those aged 16–18 years in the latter year (table 1). Overall, CCC in England and Wales declined from 501 in 2010–11 to 398 in 2011–12 (table 1), which was the lowest IMD incidence since the early 1980s (figure 1). No specific lineage was significantly associated with the decline in cases, although the largest prevalence decreases were observed in lineages 5 and 6 (table 1). The prevalence of lineages 23 and 11 increased in 2011–12, as did the frequency of isolates from genogroups C, W, and Y (table 1).

**Figure 3:**
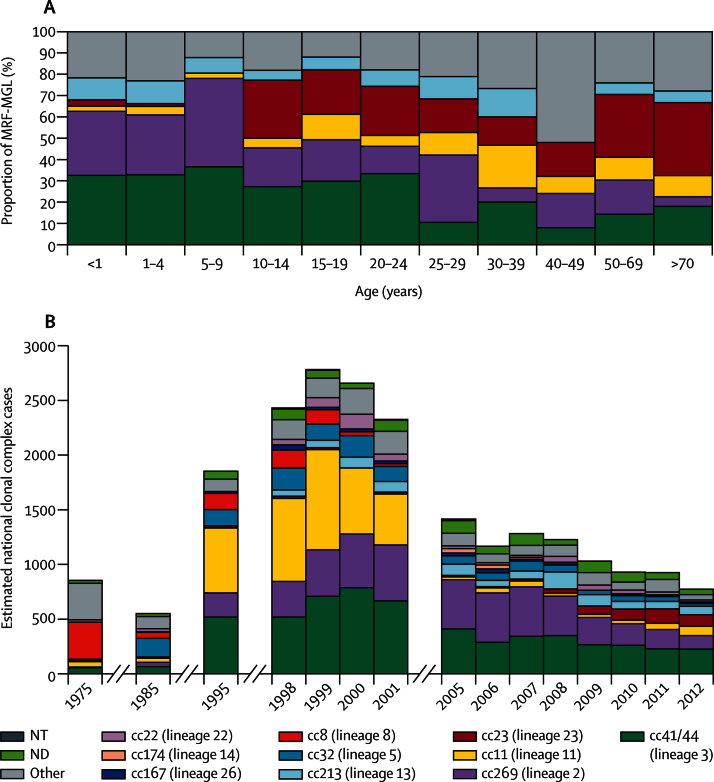
Prevalence of clonal complexes in cases of invasive meningococcal disease in England and Wales (A) Proportion of isolates for specific clonal complexes by age groups of patients in the MRF-MGL for epidemiological years 2010–11 and 2011–12. (B) Estimated yearly prevalence of major clonal complexes from 1975 to 2012. Proportions are derived from the multilocus sequence typing data available from the Public Health England Meningococcal Reference Unit^20^ multiplied by yearly disease notifications. MRF-MGL=Meningitis Research Foundation Meningococcus Genome Library. NT=multilocus sequence typing not done. ND=sequence type not designated to a clonal complex. cc=clonal complex.

We noted a strong association between patients' age and the lineage of *N meningitidis* (appendix). For example, the incidence of lineages 2 and 3 declined significantly with age (figure 3). Additionally, up to age 4 years, infection with lineage 13 *N meningitidis* was less likely than with lineage 3 *N meningitidis* (RRR 0·32, 95% CI 0·23–0·45), whereas after age 4 years patients were two orders of magnitude more likely to be infected with lineage 23 than lineage 3 (RRR 27·73 in individuals older than 25 years, 95% CI 12·91–59·56). After age 14 years, the incidence of disease caused by lineage 11 *N meningitidis* increased significantly (RRR 7·17 in individuals older than 25 years, 95% CI 3·53–14·57). Disease caused by other lineages was greatest in individuals younger than 5 years and older than 30 years, although in the latter age group lineage 3 disease remained most likely (appendix). The youngest and oldest patients were affected by the widest range of meningococcal genotypes, although four of the least diverse lineages (39, 25, 22, and 23) were often isolated from patients younger than 5 years or older than 50 years (figure 3, appendix). The association of genogroup with patients' age was consistent with the association with lineage, for example, genogroup C *N meningitidis* were more frequently identified in individuals aged 25–49 years (average 2·2 isolates per 2 year age group [11%]) than any other genogroup (average 1·5 isolates per 2 year age group [5%]; p<0·0001).

Vaccine antigen peptide sequences were diverse within the MRF-MGL. Despite possessing the greatest numbers of rare peptide subvariants, fHbp and NHBA were reasonably well conserved at the peptide sequence level (average *p* distances 0·20 [80% aminoacid identity] and 0·13 [87% identity], respectively, table 2). PorA variable region 2 was highly variable (38% aminoacid identity), with 55 different peptide subvariants identified. The NadA antigen was absent in 797 MRF-MGL isolates (89%; table 2). Vaccine antigen variants were associated with particular lineages; for example, fHbp peptide 1 (variant 1.1) was found mainly in lineage 5 isolates (appendix).^4^ Exact aminoacid sequence matches to any vaccine antigen variant occurred in 259 isolates (29%) from eight lineages. Among these, 247 (37%) of the 668 genogroup B isolates contained at least one exact match to a vaccine variant. Lineage 3 was most likely to contain an exact match to any vaccine antigen variant (203 isolates), and only one lineage 2 isolate contained an exact match. Almost a third of isolates from patients younger than 1 year (62 [31%] of 203) contained exact peptide matches to any variant, which is consistent with the association of lineage 3 with this age group (figure 3).

## Discussion

WGS technology permits genetic characterisation of bacterial isolates at more than 95% of their genomic loci, enabling unification of clinical, epidemiological, and microbiological data.^15^ To affect the care of patients, however, these data have to be provided in an accessible format that can be readily interpreted by medical and public health practitioners. The MRF-MGL achieves this for a coherent national collection of IMD isolates by presenting WGS data in an open-access web interface with in-built reporting, analysis, and export options. The infrastructure in the *Neisseria* Sequence Typing database is used to consolidate and extend the comprehensive case ascertainment data and specimen collections available in England and Wales, where virtually all IMD isolates are referred to the PHE-MRU.^16^, ^19^ Increasing volumes of WGS data will enable formal definition of the high-resolution population structure of *N meningitidis*. These data will be of particular use because of the changes in the UK meningococcal vaccine schedule, including vaccines that target serogroups A, C, W, and Y, and serogroup B meningococci.

Consistent with previous studies, IMD isolates in the MRF-MGL were genetically and antigenically diverse, with extensive evidence for the role of recombination in generating this diversity.^25^ Gene-by-gene analyses^20^ rapidly and effectively organised this diversity into coherent groups that were consistent with meningococcal clonal complexes.^2^, ^15^, ^26^ We noted good agreement between different analytical approaches, which identified at least 20 distinct meningococcal lineages. The lineage nomenclature is searchable within the *Neisseria* Sequence Typing database and the MRF-MGL, which enables comparisons of clonal complexes and other designations. In common with other geographical regions, IMD incidence in England and Wales has fluctuated,^5^, ^27^ and, using the in-built analysis tools, the MRF-MGL data could be readily placed in the context of previous studies.^28^ The largest epidemics in the past 100 years can be attributed to the disruptions of the World Wars and were probably caused by single lineages. By contrast, the increase in disease incidence seen from 1985 to 2005 was due to concurrent and successive IMD epidemics caused by lineages 5, 11, 2, and 3. These observations support the view that later IMD epidemics have been due to interactions between meningococcal diversity, human social behaviour, and host immunity. Continued surveillance using WGS data will further define the *N meningitidis* lineages, serogroups, and genogroups responsible for fluctuations in incidence and provide the genetic resolution necessary to detect outbreaks and future trends in disease. Such data will provide the basis for development of targeted interventions.

The pronounced associations between specific lineages and disease in particular age groups were consistent with those reported for pan-European MLST data from the early 2000s.^23^ The diversity of IMD isolates was increased in the oldest and youngest age groups, perhaps due to reduced immunity in these populations. We noted two important differences in patterns, that fewer lineage 11 isolates and more lineage 23 isolates were present in the MRF-MGL than in the previous European data. The difference in lineage 11 serogroup C prevalence is attributable to the introduction of meningococcal serogroup C conjugate vaccines, but the reasons for changes in the prevalence of lineage 23 serogroup Y *N meningitidis*, affecting mainly older individuals, are unclear.^27^, ^29^ Identification of such associations alongside WGS data presents the prospect of investigating the genomic basis of meningococcal phenotypes, exemplified by the over-representation of the meningococcal disease-associated phage in lineages with a propensity to cause disease in adolescents.^30^ Comparison of WGS data from invasive and carried *N meningitidis* will improve such studies.

An important advantage of WGS is the high-resolution designation of isolate relationships within lineages, such as being able to distinguish sublineage 11.1 from 11.2, which is not possible with MLST.^7^ Sublineages often show distinct epidemiological behaviours, with epidemics being attributable to specific sublineages. As more WGS data become available, the role of sublineages will become increasingly apparent, especially for those not resolvable by MLST. For example, lineage 11.1 isolates were predominant among global IMD isolates in the mid-20th century, but from the mid-1990s to early 2000s, serogroup C lineage 11.2 was the main cause of serogroup C disease in many countries, including the UK.^7^ This outbreak was successfully contained by herd immunity generated with meningococcal serogroup C conjugate polysaccharide vaccines,^3^, ^5^ but 10–14 years after implementation some lineage 11.2 serogroup C disease is recorded in the MRF-MGL, perhaps because of waning immunity in adolescents.^31^ Serogroup C lineage 11.2 *N meningitidis* has been associated with IMD outbreaks in several communities of men who have sex with men.^32^, ^33^ Finally, serogroup W lineage 11.1 *N meningitidis* was introduced into England and Wales in the early 2000s as part of the Hajj-associated outbreaks,^5^ but a later increase in lineage 11.1 serogroup W *N meningitidis* is due to a closely related but distinct variant.^24^

With no serogroup B conjugate polysaccharide vaccines, protection against serogroup B IMD depends on protein-based vaccines. Bexsero was introduced into the UK immunisation programme in late 2015. This vaccine contains an outer-membrane vesicle and one protein antigen (NHBA) from a lineage 3 *N meningitidis* with two protein antigens (fHbp and NadA) from a lineage 5 organism.^4^ Peptide sequences of vaccine antigens are readily deduced within the MRF-MGL, enabling the potential coverage of actual and proposed vaccine formulations to be assessed. Exact peptide matches to the vaccine components were rare in the MRF-MGL, especially in isolates from individuals younger than 1 year, which shows the importance of immunological cross-reactivity between peptide variants to achieve good coverage with vaccines. Cross-protection between fHbp variant 1 subvariants and NadA-1, NadA-2, and NadA-3 subvariants has been described.^34^ Potentially cross-protective fHbp subvariants were identified in 497 (55%) and NadA subvariants in 97 (11%) of MRF-MGL isolates. Therefore, 567 (63%) of the MRF-MGL, 498 (75%) of genogroup B isolates, and all 35 of the lineage 11.1 genogroup W isolates, had at least one peptide sequence match to one of the vaccine antigens, which suggests that coverage is similar to that predicted with the meningococcal antigen typing system assay,^35^ but remains subject to several assumptions. Over time, the fluctuations in IMD-associated meningococcal lineages and their antigen variants identified with WGS surveillance might warrant the development of alternative vaccine formulations.

Real-time genomic analyses of *N meningitidis* from individuals who have developed disease are increasingly feasible as technology develops: combined with the 4 day turnaround for WGS determination, the gene-by-gene approach allows genome-level characterisation of multiple isolates and comparisons with reference databases within hours.^7^ Furthermore, WGS involves the processing of individual specimens from which multiple types of information can be extracted, including high-resolution genetic relatedness of isolates, variation of vaccine and typing antigens, and likely vaccine and antimicrobial susceptibility.^36^ The delivery of these data through easily accessible interfaces will improve the ability to intervene in all bacterial infections from the level of the individual patient to entire populations. In the case of the *N meningitidis*, this will be especially important during the introduction of novel vaccines.

For the **European Nucleotide Archive** see www.ebi.ac.uk/ena

For the **MRF-MGL *Neisseria* Sequence Typing database** see to http://PubMLST.org/neisseria/

**This online publication has been corrected. The corrected version first appeared at thelancet.com/infection on Nov 17, 2015**

## Figures and Tables

**Table 1:** Characteristics of the meningococcal isolates in England and Wales in 2010–11 and 2011–12

		**Number of isolates in 2010–11 (%)**	**Number of isolates in 2011–12 (%)**	**Ratio (2011–12: 2010–11)**	**Relative risk ratio (95% CI)**	**p value**
Total MRF-MGL*		501 (100%)	398 (100%)	0·79	1·09 (1·00–1·20)†	0·7
Lineage						
	5 (cc32)	28 (6%)	14 (4%)	0·50	0·63 (0·34–1·18)‡	0·2
	6 (cc60)	15 (3%)	5 (1%)	0·33	0·42 (0·15,1·14)‡	0·1
	23 (cc23)	60 (12%)	60 (15%)	1·00	1·26 (0·90–1·76)‡	0·2
	11 (cc11)	21 (4%)	38 (10%)	1·81	2·28 (1·36–3·82)‡	0·002
Isolates						
	From patients younger than 5 years	257 (51%)	171 (43%)	0·67	0·84 (0·73–0·97)‡	0·02
	From patients aged 16–18 years	18 (4%)	27 (7%)	1·50	1·89 (1·06–3·38)‡	0·04
Genogroup						
	Y	74 (15%)	67 (17%)	0·91	1·14 (0·84–1·54)‡	0·5
	C	13 (3%)	14 (4%)	1·08	1·36 (0·64–2·85)‡	0·5
	W	26 (5%)	30 (8%)	1·15	1·45 (0·87–2·41)‡	0·2

The changes in total number of isolates, lineages, age groups, and serogroups showing the greatest variation between years are shown. MRF-MGL=Meningitis Research Foundation Meningococcus Genome Library. cc=clonal complex.

* Total number of laboratory-confirmed cases was 1053 in 2010–11 and 766 in 2011–12 (>98% of all reported cases) and comprised 899 culture-confirmed cases and 920 non-culture-confirmed cases. The relative disease incidence ratio for 2011–12:2010–11 was 0·72 (95% CI 0·66–0·79, p<0.0001, based on population sizes from the UK Office for National Statistics).

† All laboratory-confirmed cases.

‡ Culture-confirmed cases in MRF-MGL.

**Table 2:** Prevalence of meningococcal group B vaccine antigen peptide variants in the MRF-MGL for epidemiological years 2010–11 and 2011–12

	**Number of unique peptide sequence variants**		**Mean (SD) aminoacid *p* distance across all variants present**	**Most prevalent variant**			**Presence of meningococcal group B vaccine peptide variant**		
	Prevalent (n≥10)	Rare (n<10)		Peptide variant	Number (%) from all isolates (n=899)	Number (%) from genogroup B isolates only (n=668)	Peptide variant	Number (%) from all isolates (n=899)	Number (%) from genogroup B isolates only (n=668)
fHbp	12	87	0·20 (0·13)	4	159 (18%)	159 (24%)	1.1	34 (4%)	32 (5%)
NadA	3	6	0·21 (0·20)	No value*	797 (89%)	530 (79%)	8	12 (1%)	2 (<1%)
NHBA	14	70	0·13 (0·04)	2	172 (19%)	172 (26%)	2	172 (19%)	172 (26%)
PorA VR2	15	40	0·62 (0·29)	P1.9	137 (15%)	133 (20%)	P1.4	136 (15%)	136 (20%)

MRF-MGL=Meningitis Research Foundation Meningococcus Genome Library.

* Includes genome sequences where the gene was missing, contained a frame-shift mutation, or was incompletely assembled, being interrupted by the end of a contiguous sequence.
