# Corrections

**DOI:** 10.1016/S1473-3099(15)00461-2

**Published:** 2016-01

*Hill DMC, Lucidarme J, Gray SJ, et al. Genomic epidemiology of age-associated meningococcal lineages in national surveillance: an observational cohort study.* Lancet Infect Dis *2015; **15:** 1420–28*—This Article should have been published under the CC BY creative commons license. This correction has been made to the online version as of Nov 17, 2015.

