# Supplementary material for: Genomic epidemiology of age-associated meningococcal lineages in national surveillance: an observational cohort study
Source: Lancet Infect Dis. 2015 Dec;15(12):1420–8. doi: 10.1016/S1473-3099(15)00267-4 (PMC4655307; doi:10.1016/S1473-3099(15)00267-4)
Supplement: Supplementary appendix [file mmc1.pdf]

THE LANCET Infectious Diseases

Supplementary webappendix

This webappendix formed part of the original submission and has been peer reviewed. We post it as supplied by the authors.

Supplement to: Hill DMC, Lucidarme J, Gray SJ, et al. Genomic epidemiology of age-associated meningococcal lineages in national surveillance: an observational cohort study. *Lancet Infect Dis* 2015; published online Oct 27. http://dx.doi.org/10.1016/S1473-3099(15)00267-4.

Appendix: Web supplementary information.

1. DNA preparation and whole-genome sequencing.

On receipt by the MRU, isolates were cultured on Columbia Agar plus 5% (v/v) horse blood and stored on Microbank™ Bacterial & Fungal Preservation System vials (Prolab Diagnostics, Ontario, Canada) at -80°C. Prior to DNA extraction, isolates were streak plated onto Columbia Agar plus 5% (v/v) horse blood and incubated over night at 37°C in an atmosphere containing 5% CO_2. DNA was extracted using the DNeasy Blood and Tissue kit (Qiagen) in which a sterile 1 µL inoculation loop was used to transfer and disperse (by rapid rotation between thumb and forefinger) heavy sweeps of non-confluent growth directly to a screw-capped microfuge tube containing 180 µL of buffer AL and 20 µL proteinase K. This was then incubated at 56°C with occasional vortexing for at least 2 hours. Extraction was then completed in accordance the manufacturer's DNeasy® Blood & Tissue Handbook (July 2006; Gram negative bacteria protocol) using two 75µl elution steps. DNA was transported to the Wellcome Trust Sanger Institute where standard Illumina libraries were generated using 1µg of genomic DNA sheared to 200-300bp using a Beckman FX robot. Except for twelve pilot samples which constituted a single pool and were sequenced to produce 75bp reads, 96 tagged DNA samples were pooled in an equimolar ratio for sequencing in a flowcell lane on the Illumina Hi-Seq platform, generating 100bp paired-end reads; all sequence read data passed the Sanger Institute's in-house quality control assessment.

2. Genome assembly and data upload.

FASTQ files were entered to an automated pipeline, which integrates Velvet version 1.2.01,[1] VelvetOptimiser version 2.2.0, and BIGSdb software,[2,3] for genome assembly and upload to the PubMLST *Neisseria* database (http://pubmlst.org/neisseria/).[2] No scaffolding was carried out and only contigs greater than 200bp were entered to the database. There was no manual improvement of assemblies at this stage of the project: each *de novo* assembly was assessed for the presence of core meningococcal genes and for incompletely assembled coding sequences (CDS) as defined in the PubMLST.org/neisseria sequence definitions database, an approach that produced high quality genomic data providing allele information for an average of 1,571 loci per genome (>79% of approx. 1,976 meningococcal coding sequences and >97% of 1605 core genome coding sequences)[3,4] (Table S1). Further information about the assembled genomic data for individual isolates is available in the MRF-MGL at PubMLST.org/neisseria. The allele information obtained was more than sufficient for the results presented (typing and identification of meningococcal lineages using rMLST and cgMLST). The SPAdes assembler,[5] introduced subsequent to the start of this project, also produces outputs sufficient for the analyses presented and highly similar to those of Velvet; for example, Velvet and SPAdes produce identical allele results for up to 98% of the 1,605 *N. meningitidis* core genome loci. For consistency,

all isolates were assembled using Velvet/VelvetOptimiser. For additional information on the methods chosen for processing genomic sequence data the reader is referred to Bratcher *et al, BMC Genomics*. 2014.[3]

Table S1. Summary of 2010/11 and 2011/12 automated Velvet/VelvetOptimiser genome assemblies.

Metric	Average (median)	Standard deviation
N50 contig number+	18.60	3.35
N50 contig length (L50) (bp)+	38,387	6,408
Assembly mean contig length (bp)	10,657	1,727
Largest contig length	121,962 (115,208)	37,572
Number of contigs	209	39
Number of contigs >1000 bp	114	17
Total assembled bases	2,166,197	44,680
Total assembled bases in contigs >1000 bp	2,128,374	40,019
Velvet final assembly *k*-mer size	85	5
Approx. number completely assembled core-genome CDS†	1,571	10
%GC	51.60	0.15
Estimated sequencing coverage*	172.64	97.38
Estimated contig coverage‡	0.99	0.02

+N50 contig number: number of contigs that collectively cover at least 50% of the assembly; N50 contig length (L50): 50% or more of the genome present on contigs of ≥Nbp. †Core-genome CDS: 1,605 coding sequences (CDS) present in >95% of *N. meningitidis* genomes.[3] *Estimated sequencing coverage: number of bases in sequence reads as a proportion of final assembly size. ‡Estimated contig coverage: number of assembled bases as a proportion of FAM18 reference genome (2,194,961bp).[4]

3. Genome annotation.

Where the locus allelic variant was already stored in the database, or where BLAST hits were within 98% identity and alignment to database stored alleles, annotation of genomes with loci and allele numbers was automatically carried out by the BIGSdb ‘autotagger’ and ‘autodefiner’ tools. Where there was no annotation for a locus within an isolate web-based sequence tagging[6] was used for manual curation: BLAST hits were extracted and aligned in the MEGA (version 6.0) software package[7] for the upload of new alleles to the database, or, missing loci and those interrupted by the end of a contig within isolates were assigned alleles ‘0’ and ‘s’ respectively**.**

4. Meningococcal lineages in MRF-MGL.

Congruence between clonal complexes and rMLST clusters (figure 2A; Table S2) was assessed by calculating adjusted Wallace Coefficients (AW)[8] using the online tool at www.comparingpartitions.info. rMLST clusters were congruent

with isolate MLST clonal complexes, with the exception of the rST from isolate ID 21505 (cc103) (figure 2A; table S2). Although the three lineage 2 (cc269) rMLST clusters (figure 2A) were in general composed of distinct STs (Table S5), their relationships were inconsistent with those of seven-locus MLST and cgMLST clusters (figure 2B; figure S6; figure S7). This was likely due to recombination within some of the ribosomal protein loci that form the rMLST scheme (figure S8). Over the whole dataset, isolates from the same rMLST cluster had a 97% chance of being placed in the same clonal complex by MLST, whereas isolates of the same clonal complex had an 81% chance of belonging to the same rMLST cluster, in part reflecting the additional resolution of cc269 by rMLST ($AW_{rMLST\text{->}MLST}$ = 0.97 [0.91-0.99], $AW_{MLST\text{->}rMLST}$ = 0.81 [0.80-0.82]). At the cgMLST level (figure 2B; figure S4a), isolates belonging to the same clonal complex had a 96.7% (95% CI: 96.4-97.0) chance of being placed in the same lineage as measured using the AW statistic.

Table S2: Frequency and diversity of meningococcal disease-causing lineages, MRF-MGL 2010/11 and 2011/12

Clonal complex	Count (%) 2010/11	Count (%) 2011/12	Total count (%)	Genogroup (% if <100%)	Count unique STs (CC)	Count unique rSTs (CC)	Count incongruent rSTs (lineage)	Lineage	Count STs (lineage)	Count rSTs (lineage)	Mean pairwise rST distance (SD)
cc41/44	134 (26.75)	103 (25.88)	237 (26.36)	B (99.2), C (0.8)	81	156	0	3	84	158	11.31 (4.93)
cc269	98 (19.6)	73 (18.34)	171 (19.02)	B (98.8), C (1.2)	29	89	19 (2)*	2	50	110	11.09 (6.56)
cc23	60 (11.98)	60 (15.08)	120 (13.35)	Y	14	21	0	23	14	21	4.76 (3.29)
cc213	38 (7.58)	37 (9.30)	75 (8.34)	B	21	53	0	13	21	53	7.48 (2.88)
cc11	21 (4.19)	38 (9.55)	59 (6.56)	W (59.3), C (25.4), B (15.3)	5	16	0	11	5	16	7.27 (3.93)
cc32	28 (5.59)	14 (3.52)	42 (4.67)	B	14	23	0	5	16	24	4.62 (2.49)
cc22	13 (2.59)	11 (2.76)	24 (2.67)	W (83.0), Y (17.0)	8	15	0	22	9	16	4.95 (2.95)
cc60	15 (2.99)	5 (1.26)	20 (2.22)	B (85.0), E (15.0)	11	20	0	6	13	22	7.91 (3.43)
cc162	6 (1.20)	9 (2.26)	15 (1.67)	B	1	4	0	25	1	4	3.66 (1.75)
cc461	10 (2.00)	4 (1.01)	14 (1.56)	B	3	9	0	39	3	9	4.02 (1.99)
cc174	8 (1.60)	2 (0.50)	10 (1.11)	Y (80.0), W (10.0)	2	5	0	14	2	5	5.19 (4.80)
cc18	5 (1.00)	4 (1.01)	9 (1.00)	B	6	8	0	18	7	9	9.67 (5.68)
cc35	7 (1.40)	2 (0.50)	9 (1.00)	B	5	7	0	35	6	8	8.54 (4.35)
cc103	5 (1.00)	3 (0.75)	8 (0.89)	C (62.5), B (25.0), Y (12.5)	4	5	1 (26)	20	4	4	5.67 (1.85)
cc1157	3 (0.60)	4 (1.01)	7 (0.78)	B	5	7	0	15	5	7	3.47 (1.99)
cc167	4 (0.80)	2 (0.50)	6 (0.67)	Y	4	4	0	26	5	5	10.09 (4.54)
cc282	5 (1.00)	1 (0.25)	6 (0.67)	B	3	3	0	32	3	3	7.51 (3.44)
cc226	1 (0.20)	0	1 (0.11)	cnl	1	1	0	29	1	1	na
cc5	1 (0.20)	0	1 (0.11)	A	1	1	0	10	1	1	na
cc865	0	1 (0.25)	1 (0.11)	B	1	1	0	41	1	1	na
ccND	39 (7.78)	25 (6.28)	64 (7.12)	B (92.2), Y (3.1), C (3.1), X (1.6)	53	56	0	NA	21	21	na
**Total**	**501 (100.00)**	**398 (100.00)**	**899 (100.00)**	**B (74.21); Y (15.71); W (6.07); C (3.14); E (0.33); cnl (0.22); A, W/Y, X (0.11)**	**272**	**498**	**20**		**272**	**498**	**26.82 (8.45)**

Unique rSTs: six rSTs are found among both ccND isolates and isolates designated to a clonal complex and are counted once in the column total. Incongruent rSTs: rSTs that do not cluster into a lineage with other rSTs from isolates of the same clonal complex. *cgMLST clustered isolates possessing these rSTs into a lineage with other isolates of cc269. Mean

pairwise rST distance: the number of rMLST loci at which there is an allelic difference in a comparison between an rST pair (single occurrences of rSTs used to reduce skew). na: not applicable.

Lineages identified using nucleotide data.

Phylogenies of MRF-MGL isolates were created from rMLST and cgMLST nucleotide data (SI Fig. S3-S4) for corroboration of the lineages identified using the gene-by-gene approach (Fig. 2 & SI Fig. 4a).[9] For phylogenies created using nucleotide data, locus subsets were aligned and concatenated into single alignments using the Genome Comparator MAFFT implementation; variable sites were extracted and maximum likelihood trees were drawn using default options in MEGA6.[7] Trees were annotated using FigTree v1.4.2 (http://tree.bio.ed.ac.uk/software/figtree/). The Neighbor-Net graph was generated in SplitsTree4[10] as in the main text. The lineages and sub-lineages identified using the 49 non-paralagous rMLST or cgMLST loci, or using allele or nucleotide data, were analogous, as they were in Kohl *et al*, J Clin Microbiol 2014 for example.[11] A distance matrix generated from the core-genome loci of each of the 899 MRF-MGL genomes was rapidly calculated, but it was not practicable to display a Neighbor-Net graph due to display limitations of the SplitsTree4 program. Each genome was unique at the cgMLST level.

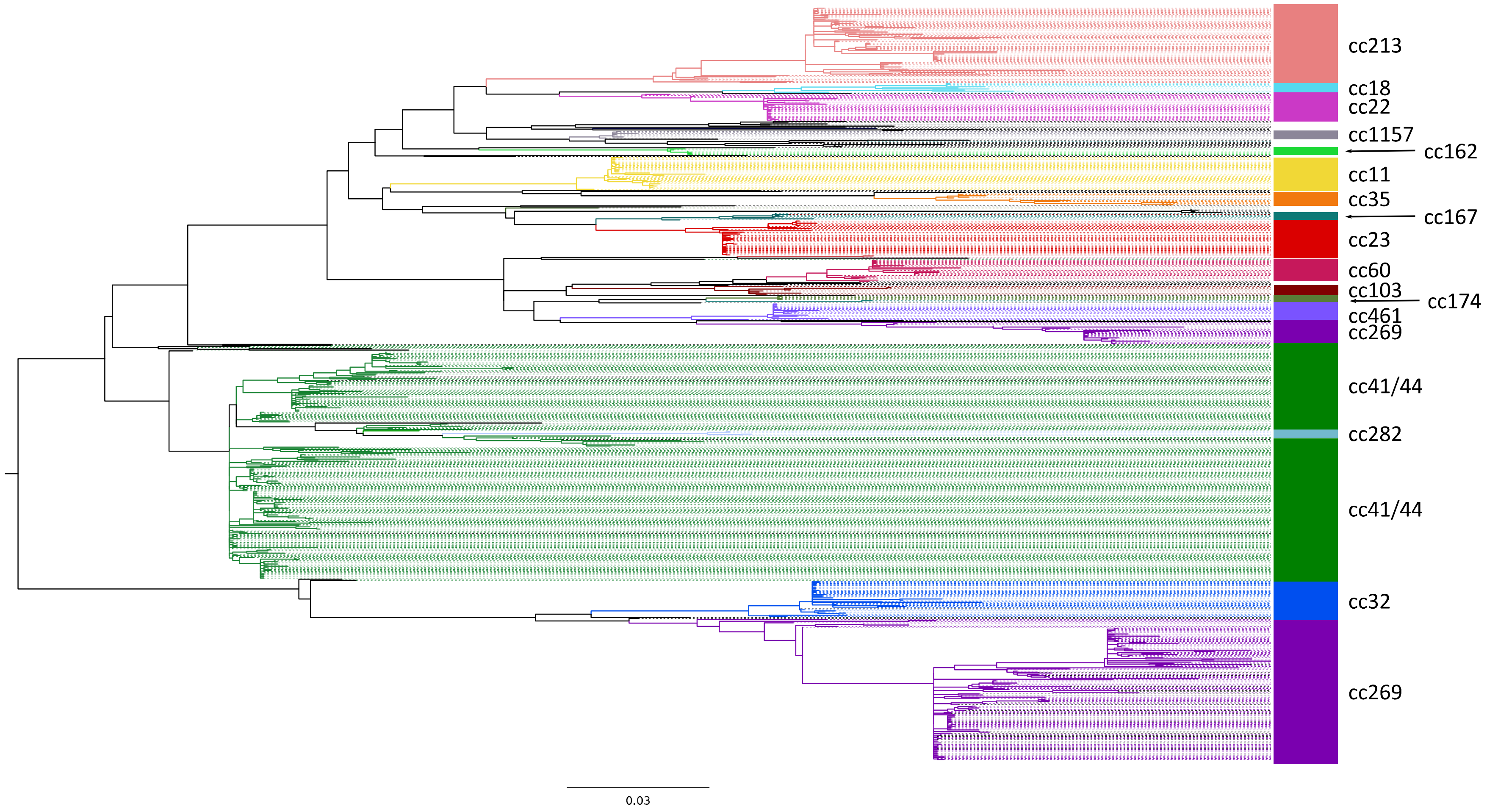

Fig. S3. rMLST variable-site maximum likelihood tree, MRF-MGL.

Nucleotide alignments of concatenated non-paralagous rMLST loci from all unique rSTs (n=498) in the MRF-MGL were extracted using Genome Comparator. A maximum likelihood tree was drawn from all variable sites (n=1,373) using MEGA6. Tips and branches are coloured by clonal complex, since lineages 3 (cc41/44) and 2 (cc269) are composed of a greater number of clusters, interspersed by other clonal complexes, in this tree compared to phylogenies created using allele data (Figure 2A) or core genome loci (Fig. S4). This indicates conflicting signal in the data, as a result of horizontal gene transfer, when compared to all the other phylogenies.

a)

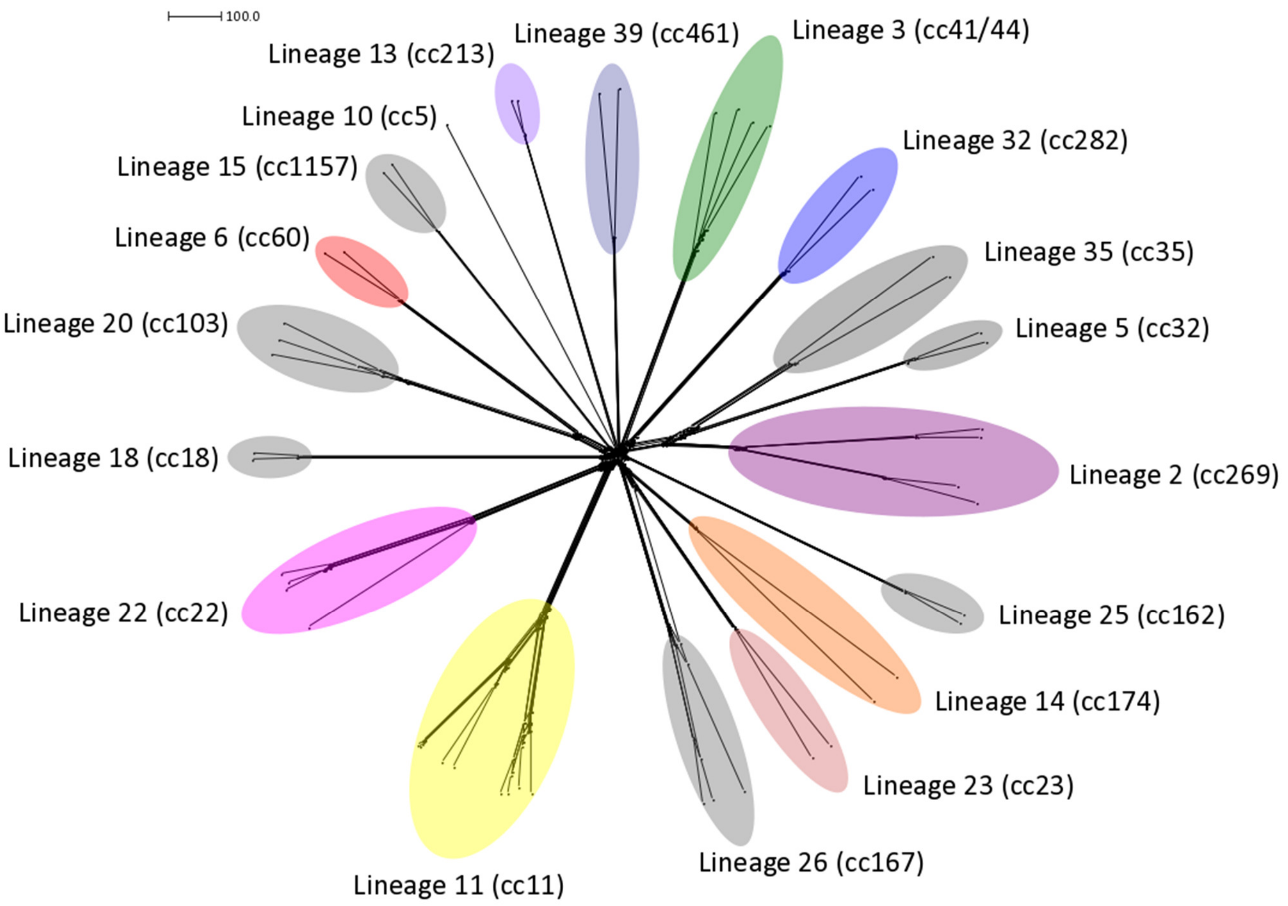
100.0
Lineage 13 (cc213)
Lineage 39 (cc461)
Lineage 3 (cc41/44)
Lineage 10 (cc5)
Lineage 32 (cc282)
Lineage 15 (cc1157)
Lineage 6 (cc60)
Lineage 35 (cc35)
Lineage 20 (cc103)
Lineage 5 (cc32)
Lineage 18 (cc18)
Lineage 2 (cc269)
Lineage 22 (cc22)
Lineage 25 (cc162)
Lineage 14 (cc174)
Lineage 23 (cc23)
Lineage 11 (cc11)
Lineage 26 (cc167)

b)

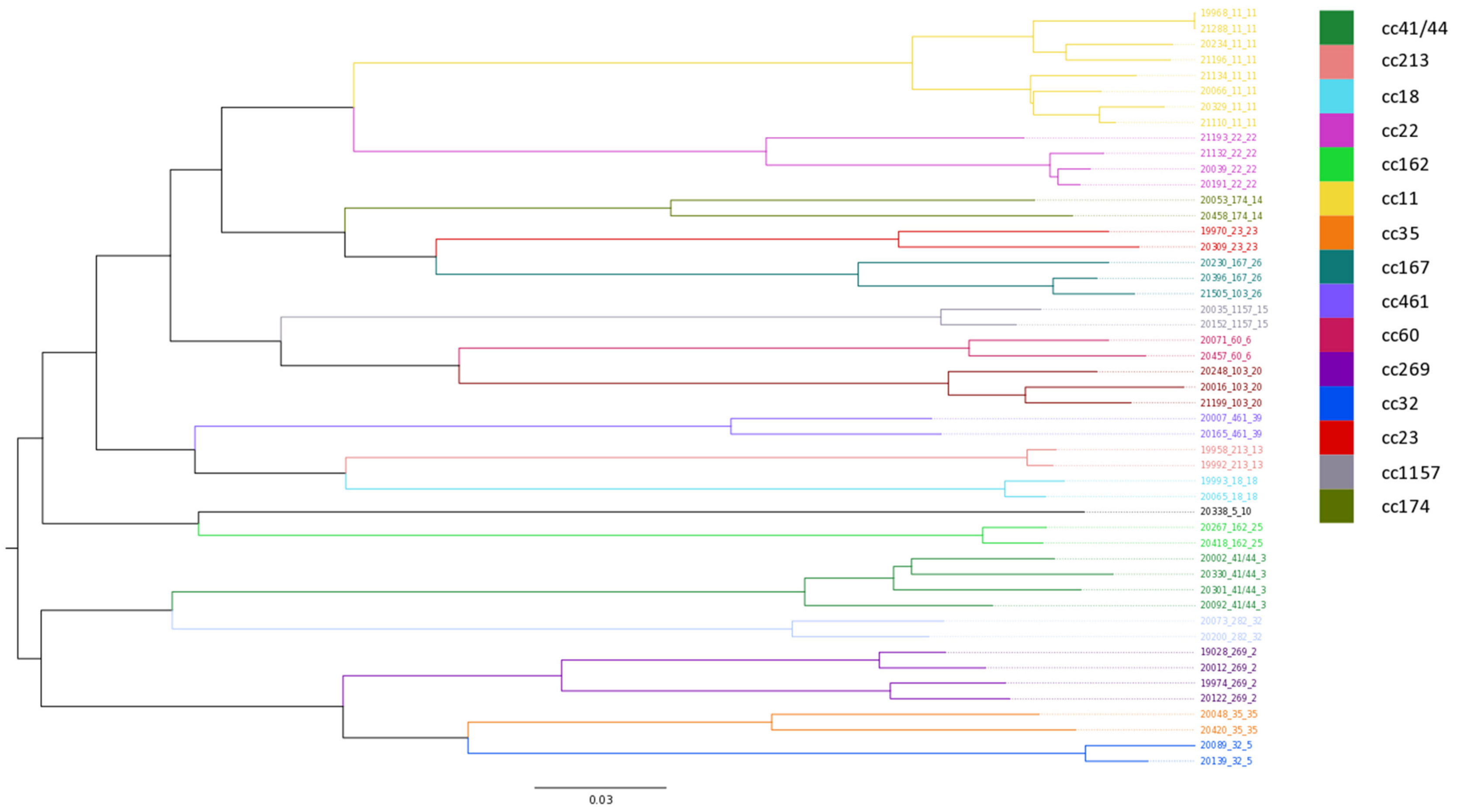

Fig. S4. MRF-MGL lineages identified using core-genome loci.

A subset of isolates (n=50) representing each lineage in the MRF-MGL was extracted to demonstrate that core-genome lineages, inferred using allele or nucleotide data, are analogous to those inferred using rMLST loci (Figure 2A). a) Neighbor-Net graph generated from allelic distances among core-genome loci. b) Maximum likelihood tree generated from variable sites (n=104,993) in the concatenated core-genome locus nucleotide alignment.

Lineage 2 sub-lineages.

Table S5. Association of MRF-MGL MLST sequence types (ST) with lineage 2 sub-lineages.

Lineage 2 sub-lineage (cgMLST)	MLST ST	rMLST cluster
2.1	269	1 (and single rST in 3)
	1049	1
	1195	1
	1092	1
	1942	1
	2873	1
	7226	1
	7939	1
	9823	1
	9836	1
	9840	1
	9843	1
	467	3
	479	3
	283	3
	1774	3
	10264	3
	10291	3
2.2	1161	2
	275	2
	1163	2
	4713*	2
	1159*	2
	6604	2
	5849*	2
	1831*	2
	4401	2
	6428	2
	7789	2
	7833	2
	9004	2
	9826	2
	9829	2
	10288	2
	5335*	2
	6781*	2
	7143*	2
	9839*	2
	9880*	2
	9837*	2
	3934*	2
	2307*	2
	9827*	2
	9887*	2
	9845*	2
	10265*	2
	10290*	2
	10263*	2
	10297*	2
	10283*	3
Putative 2.3	13	
	9311*	2

* indicates sequence types not designated to MLST-defined clonal complexes, but which would be part of cc269/275 if the second central genotype were incorporated.

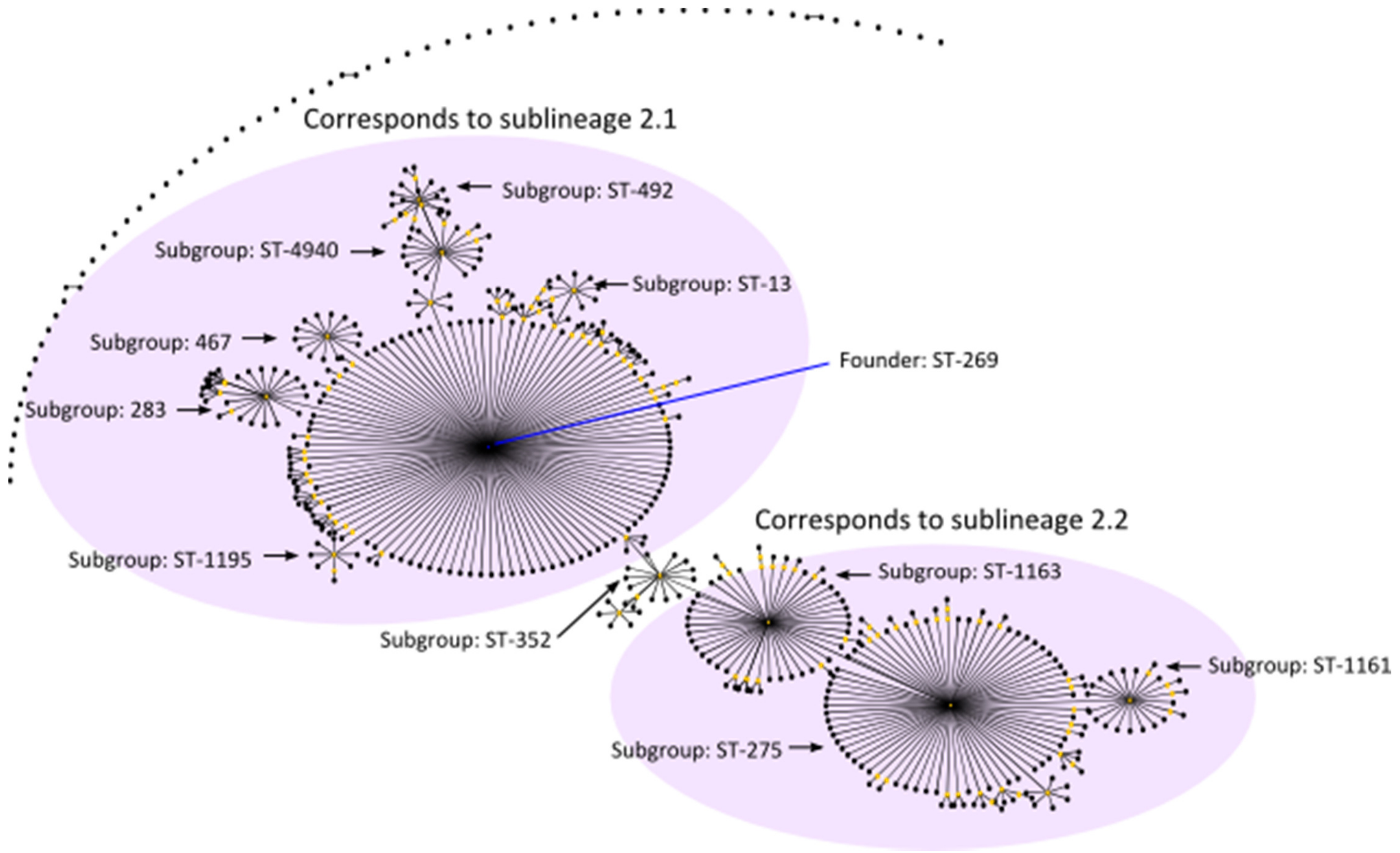

Fig. S6. MLST eBURST of PubMLST/neisseria sequence types (ST) associated with lineage 2.

cc269-designated STs, and STs not designated to MLST-defined clonal complexes that would be part of cc269/275, were included in the eBurst[12] diagram. STs are represented as coloured circles: the founding ST is blue, subgroup founding STs are yellow, and other STs are black. Each ST was included once, and sub-groups were labelled according to their founders if the founder was prevalent in PubMLST.org/neisseria. The approximate clustering of lineage 2.1 and 2.2 isolates as identified in Figure 2B is indicated with purple shading.

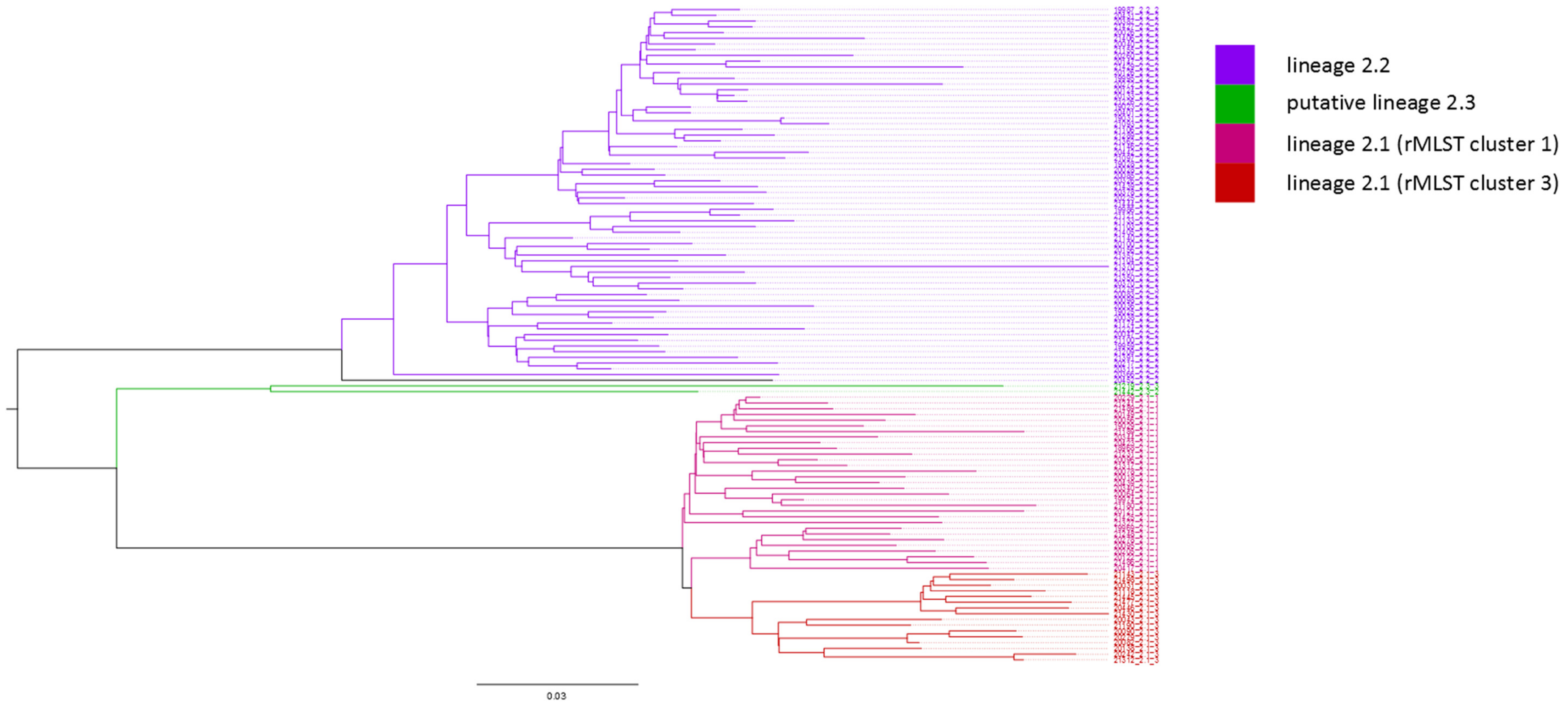

Fig. S7. Lineage 2 core-genome maximum-likelihood tree

A subset of lineage 2 (cc269) isolates (n=116) representing each lineage 2 sub-lineage in the MRF-MGL was chosen to demonstrate that sub-lineages identified by the rMLST gene-by-gene approach (Figure 2B) are congruent to those in a traditional maximum likelihood tree. Variable sites (n=79,316) were extracted from the concatenated alignment of core-genome loci. As in Figure 2B, lineage 2.1 is composed of rMLST clusters 1 and 3 (Figure 2A, Table S5) due to a probable recombination event in the rMLST loci (Fig. S8).

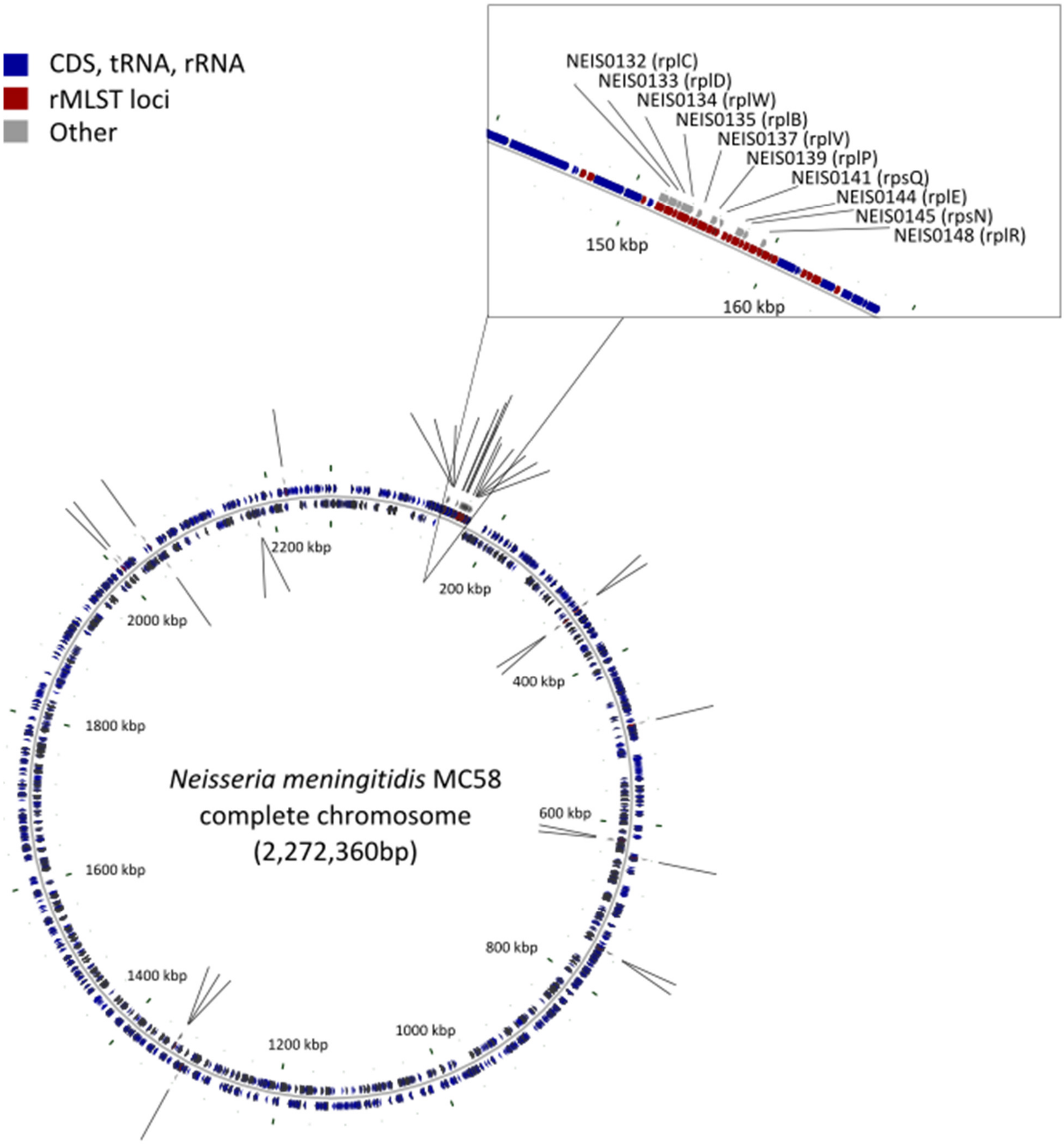

Fig. S8. Ribosomal MLST loci responsible for incongruence between rMLST and MLST/cgMLST lineage 2 network topologies.

Neisseria meningitidis genes are indicated by triangles on the MC58 chromosome, with gene product indicated by colour. *N. meningitidis* rMLST loci are additionally indicated by grey tick marks. Zoom panel: rMLST loci that distinguish rMLST sub-cluster 3 from rMLST sub-clusters 1 and 2 are annotated by name on grey tick marks. This figure was generated with CGView.[13]

5. Meningococcal lineages associated with patient age groups.

Multinomial regression analysis of meningococcal disease-causing lineage on patient age was carried out with lineages modelled as categorical variables (with 'lineage 3 (cc41/44)' the baseline outcome) in the 'nnet' package with the function 'multinom'.[14] Patient age was grouped into four categories that reflected the peaks in meningococcal disease incidence in infants and adolescents and low incidence in the elderly.[15] This analysis indicated strong, apparently non-linear, associations between the age of the patient and the relative risk of disease from particular lineages (Table S9). Additional analyses with alternative cut-offs/bands for patient age that retained the relative peaks in incidence (e.g. <2, 2-12, 13-28, >28),[16] produced the same general trends.

Table S9. Multinomial regression of meningococcal lineage against patient age group, baseline outcome lineage 3 (cc41/44).

Lineage	Age (yr)	# isolates	Relative risk ratio	95% confidence intervals
2	≤4	124	0.89	0.70, 1.13
2	5-14	21	1.13	0.59, 2.17
2	15-24	18	0.62	0.33, 1.15
2	≥25	35	0.86	0.52, 1.42
**23**	**≤4**	**9**	**0.06**	**0.03, 0.13**
**23**	**5-14**	**6**	**4.44**	**1.44, 13.76**
**23**	**15-24**	**23**	**10.84**	**4.59, 25.59**
**23**	**≥25**	**82**	**27.73**	**12.91, 59.56**
**13**	**≤4**	**45**	**0.32**	**0.23, 0.45**
13	5-14	4	0.59	0.19, 1.82
13	15-24	7	0.66	0.27, 1.59
13	≥25	18	1.22	0.64, 2.31
**11**	**≤4**	**14**	**0.10**	**0.06, 0.17**
11	5-14	2	0.95	0.20, 4.49
**11**	**15-24**	**10**	**3.03**	**1.24, 7.42**
**11**	**≥25**	**33**	**7.17**	**3.53, 14.57**
**other**	**≤4**	**96**	**0.69**	**0.53, 0.89**
other	5-14	9	0.62	0.27, 1.42
other	15-24	15	0.66	0.34, 1.29
**other**	**≥25**	**83**	**2.63**	**1.69, 4.10**

Risk ratios are presented relative to the risk of lineage 3 disease in each age category ('baseline outcome' was lineage 3 in this model). Significant relative risk ratios in bold.

7. Bexsero® antigens in meningococcal lineages

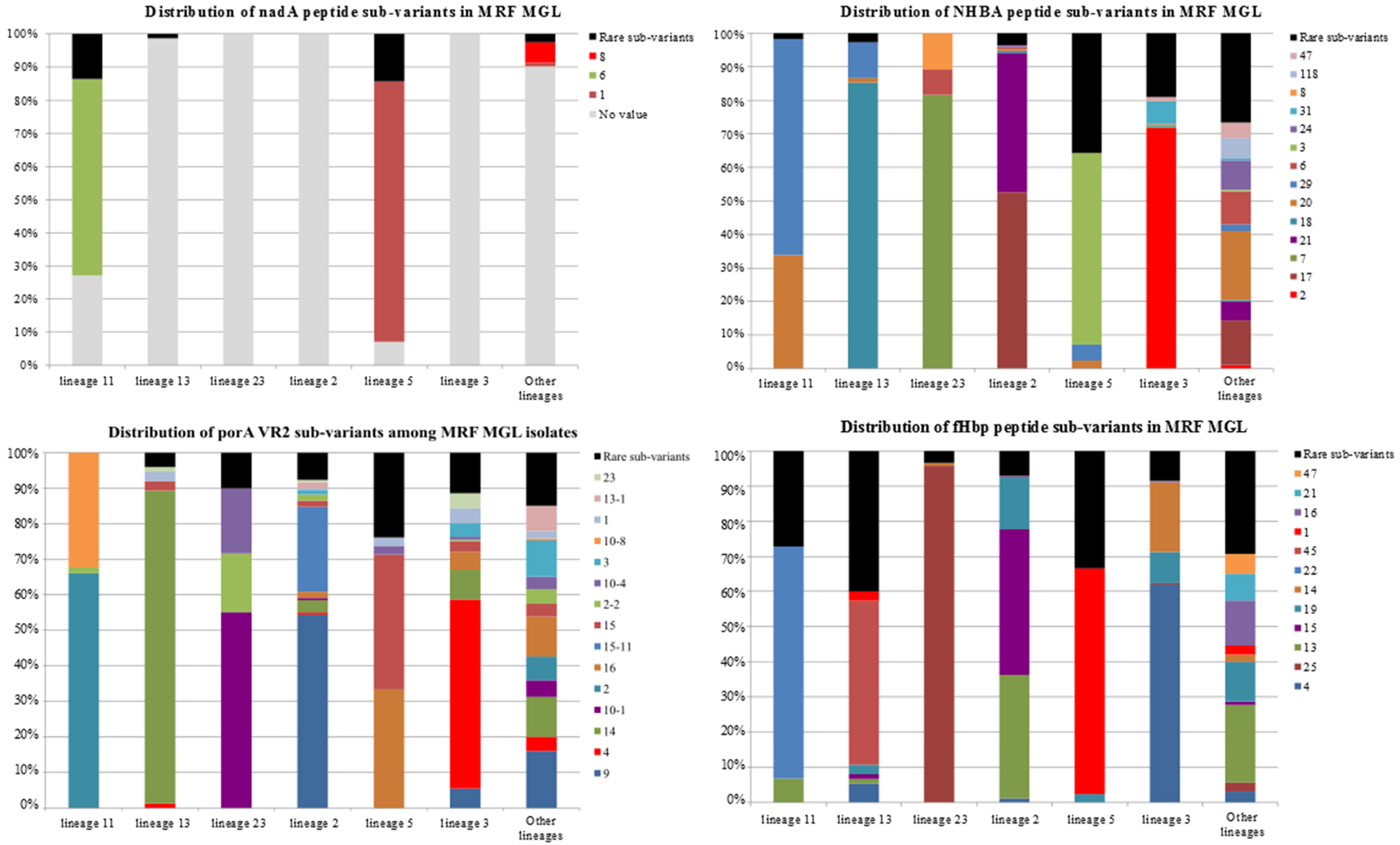

Distribution of nadA peptide sub-variants in MRF MGL
Rare sub-variants
8
6
1
No value
lineage 11
lineage 13
lineage 23
lineage 2
lineage 5
lineage 3
Other lineages
Distribution of NHBA peptide sub-variants in MRF MGL
Rare sub-variants
47
118
8
31
24
3
6
29
20
18
21
7
17
2
Distribution of porA VR2 sub-variants among MRF MGL isolates
Rare sub-variants
23
13-1
1
10-8
3
10-4
2-2
15
15-11
16
2
10-1
14
4
9
Distribution of fHbp peptide sub-variants in MRF MGL
Rare sub-variants
47
21
16
1
45
22
14
19
15
13
25
4

Fig. S10. Bexsero© Vaccine antigen peptide variants in meningococcal isolates from culture-confirmed cases of meningococcal disease in England and Wales, 2010/11-2011/12. Distribution of vaccine antigen peptide sequence variants (fHBP, NHBA, NadA, and PorA VR2) among isolates, grouped by lineage. 'Rare' variants are those present in fewer than 10 isolates. The peptide variant included in the Bexsero® (Novartis) formulation is coloured red. 'No value' refers to absence of the peptide (the result of missing, frame-shifted, or incompletely assembled antigen genes.

References

1 Zerbino DR, Birney E. Velvet: algorithms for de novo short read assembly using de Bruijn graphs. *Genome Res*. 2008; **18**(5): 821-9.

2 Jolley KA, Maiden MC. BIGSdb: Scalable analysis of bacterial genome variation at the population level. *BMC Bioinformatics*. 2010; **11**(1): 595.

3 Bratcher HB, Corton C, Jolley KA, Parkhill J, Maiden MC. A gene-by-gene population genomics platform: *de novo* assembly, annotation and genealogical analysis of 108 representative *Neisseria meningitidis* genomes. *BMC Genomics*. 2014; **15**: 1138.

4 Bentley SD, Vernikos GS, Snyder LA, et al. Meningococcal genetic variation mechanisms viewed through comparative analysis of serogroup C strain FAM18. *PLoS Genet*. 2007; **3**(2): e23.

5 Bankevich A, Nurk S, Antipov D, et al. SPAdes: a new genome assembly algorithm and its applications to single-cell sequencing. *J Comput Biol*. 2012; **19**(5): 455-77.

6 Jolley KA, Maiden MC. Automated extraction of typing information for bacterial pathogens from whole genome sequence data: *Neisseria meningitidis* as an exemplar. *Euro Surveill*. 2013; **18**(4): 20379.

7 Tamura K, Stecher G, Peterson D, Filipski A, Kumar S. MEGA6: Molecular Evolutionary Genetics Analysis version 6.0. *Mol Biol Evol*. 2013; **30**(12): 2725-9.

8 Severiano A, Pinto FR, Ramirez M, Carriço J. Adjusted Wallace as a Measure of Congruence between Typing Methods. *J Clin Microbiol*. 2011.

9 Maiden MC, van Rensburg MJ, Bray JE, et al. MLST revisited: the gene-by-gene approach to bacterial genomics. *Nat Rev Microbiol*. 2013; **11**(10): 728-36.

10 Huson DH. SplitsTree: analyzing and visualizing evolutionary data. *Bioinformatics*. 1998; **14**(1): 68-73.

11 Kohl TA, Diel R, Harmsen D, et al. Whole-genome-based *Mycobacterium tuberculosis* surveillance: a standardized, portable, and expandable approach. *J Clin Microbiol*. 2014; **52**(7): 2479-86.

12 Feil EJ, Li BC, Aanensen DM, Hanage WP, Spratt BG. eBURST: inferring patterns of evolutionary descent among clusters of related bacterial genotypes from multilocus sequence typing data. *J Bacteriol*. 2004; **186**(5): 1518-30.

13 Stothard P, Wishart DS. Circular genome visualization and exploration using CGView. *Bioinformatics*. 2005; **21**(4): 537-9.

14 Venables WN, Ripley BD. Modern Applied Statistics with S. 4th ed. New York: Springer; 2002.

15 Brehony C, Trotter CL, Ramsay ME, et al. Differential age distribution of disease-associated meningococcal lineages-Implications for vaccine development. *Clinical and Vaccine Immunology*. 2014.

16 Bille E, Ure R, Gray SJ, et al. Association of a bacteriophage with meningococcal disease in young adults. *PLoS ONE*. 2008; **3**(12): e3885.
